# Hydraulic Asymmetries for Biological and Bioinspired Valves in Tubular Channels: A Numerical Analysis

**DOI:** 10.3390/biomimetics11020087

**Published:** 2026-01-26

**Authors:** Francesco Varnier, Reza Norouzikudiani, Giovanni Corsi, Daniele Agostinelli, Ido Levin, Antonio DeSimone

**Affiliations:** 1 The BioRobotics Institute, Scuola Superiore Sant’Anna, Viale R. Piaggio 34, 56025 Pontedera, Italy; francesco.varnier@santannapisa.it (F.V.); reza.norouzikudiani@santannapisa.it (R.N.);; 2 DIISM, Università Politecnica delle Marche, Via Brecce Bianche 12, 60131 Ancona, Italy; 3 UBC Chemistry, University of British Columbia, Vancouver, BC V6T 1Z1, Canada; ido.levin@ubc.ca; 4 MathLab, SISSA-International School for Advanced Studies, Via Bomomea 265, 34136 Trieste, Italy

**Keywords:** Tesla valve, bioinspired, diodicity, computational fluid dynamics, COMSOL Multiphysics, numerical simulation

## Abstract

Biological, biomimetic, and engineering systems make extensive use of hydraulic asymmetries to control flow inside tubular structures. Examples span physiological valves, the guided transport observed in shark intestines, and passive devices such as Tesla valves. Here we investigate the mechanisms that generate these asymmetries using the notion of diodicity, defined as the ratio between pressure drops required to drive the same flow in opposite directions. We first focus on 2D geometries, which allow us to identify and study the main contributions to hydraulic asymmetry: channel geometry and internal obstacles embedded within a channel with rigid walls. By considering both rigid and deformable obstacles, we model channels that always remain open in both directions and channels that can be completely blocked by valve-like structures. We then extend the analysis to 3D geometries, again considering rigid and elastic cases. As a general trend, we find that geometry alone establishes a baseline diodicity, while higher dimensionality and structural reconfiguration consistently amplify the effect.

## 1. Introduction

In many natural and engineered systems, fluid flow through a channel depends on the direction of motion. The same geometry can offer low resistance in one direction and much higher resistance in the opposite one. We refer to this directional contrast in hydraulic response as hydraulic asymmetry.

**Figure 1:**
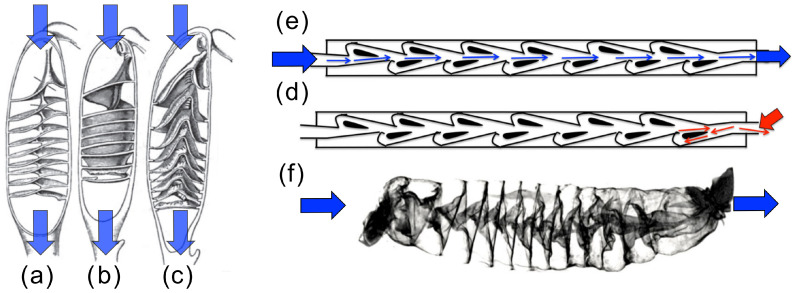
On the left, sketches of shark intestine representing different geometries: (**a**) columnar, (**b**) funnels pointed posteriorly, and (**c**) funnels pointed anteriorly. The three geometries represent different designs generating hydraulic asymmetry. Blue arrows indicate the preferential direction. On the right, a direct comparison with a Tesla valve: flux in forward direction occurs preferentially along the central axis (blue arrow) (**e**), while flux in the opposite direction (red arrow) involves significant recirculation (**d**). The similarity between this device and the shark intestine is clearly visible in (**f**). Images taken from [3].

**Figure 2:**
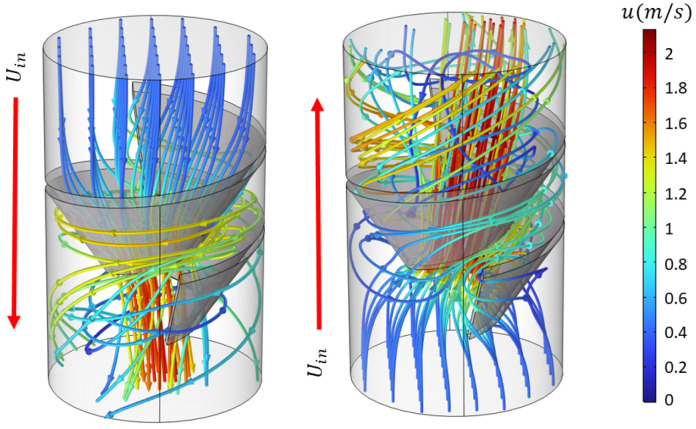
Numerical streamlines of flow through a channel containing a spiral obstacle inspired by shark intestines. Differences between forward and reverse flow directions demonstrate the inherent hydraulic asymmetry.

Biological organisms make extensive use of this principle. A clear example is provided by heart valves made of thin leaflets that swing open under the forward pressure pulse and fold closed as soon as the pressure drops, sealing the passage and preventing significant backflow. Similarly, the lymphatic system contains intraluminal leaflet valves that function to bias lymph flow back towards the heart [1]. Exploiting the same concept with a different strategy, sharks achieve hydraulic asymmetry without occlusion in their digestive system. The intestines of sharks contain a segment called the spiral intestine, which consists of a tubular structure with internal spiral and funnel-like segments. Interestingly, this structure was demonstrated to steer the flow preferentially along the digestive path and hinder motion in the opposite direction (Figure 1). This mechanism is reproduced numerically in Figure 2, where streamlines in a channel with a rigid spiral obstacle inspired by the intestine differ markedly between forward and reverse flow. Experimental exploration of simplified spiral-intestine-like structures demonstrates that this geometry indeed induces hydraulic asymmetry, and highlights the effect of channel deformability [2].

Engineered devices reproduce these ideas, often inspired by nature. Tesla valves [4] guide the flow through branches and bends that, as in the shark intestine, favor one direction over the other without any moving parts. Recent Tesla-valve applications are reviewed in [5]. Tesla-type channels have been used to suppress backflow in micro- and nanofluidic pumping architectures [6,7,8], and to enhance mixing by exploiting their reverse-flow response [9,10], including a multistage food-science demonstration (litmus reagent and vinegar) [11]. Related layouts have been proposed for thermal management, such as lithium-ion battery cooling [12], and for energy devices such as fuel cells [13] and photobioreactors [14]. These studies include numerical simulations to calculate and optimize diodicity values (see [15,16,17,18]).

Deformable channels have been considered in the literature, especially with reference to the possibility of tuning the diodicity properties by applying variable external stresses to the conduits [19]. For further work on deformable channels for microfluidic applications, we also refer the reader to [20,21].

**Figure 3:**
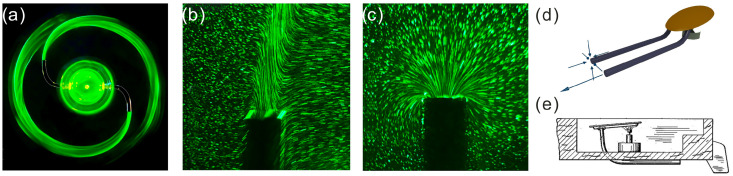
(**a**–**c**): Feynman sprinkler [22]. (**a**): Streakline photograph of the sprinkler operating in forward mode, using fluorescein dye. (**b**): Detail of the emitted jet and the resulting path lines near the outlet in forward operation. (**c**): Detail of the suction flow near the outlet in reverse operation. (**d**,**e**): Putt-Putt boat. (**d**): Back view of the chamber and exhaust tubes illustrating the expulsion–suction cycle. (**e**): Schematic diagram of a diaphragm-type Putt-Putt engine (based on a 1934 patent by Paul Jones), where asymmetric expulsion and re-entry of water generate net thrust. In both systems, the streamline topology differs between the two directions, illustrating a geometric origin of hydraulic asymmetry. Panels (**a**–**c**) reproduced and adapted from [22], with permission of the authors.

Beyond static channels, hydraulic asymmetries can generate propulsion. In the Feynman sprinkler (Figure 3), expulsion and suction involve different streamline patterns [22]. A similar mechanism operates in the Putt-Putt boat: asymmetric expulsion and re-entry of water through the same tubes lead to unequal forces on the fluid and, hence, net thrust [23].

Across all these biological and technological examples, the same feature emerges: the same pressure difference can generate different flow rates and streamline patterns when the flow is reversed. We refer to this lack of odd symmetry as hydraulic asymmetry. By contrast, odd symmetry is to be expected for flows in undeformable domains at very low Reynolds numbers, because of the linearity of the governing Stokes equations, and for flows in undeformable domains with fore-aft symmetry.

The goal of this paper is to contribute to the quantitative understanding of the mechanisms underlying hydraulic asymmetry. In particular, we aim to clarify how much asymmetry can arise from geometry alone, how deformable obstacles modify or amplify it, and whether trends observed in simplified 2D settings persist in three-dimensional channels.

The key contributions and main findings of this work are as follows. First, in rigid channels with obstacles that are not fore-aft symmetric, inertial effects lead to different geometries of the streamlines when the flow direction is reverted. Thus, geometry alone is sufficient to establish baseline levels of hydraulic asymmetry. Second, larger differences in forward versus backward flow are generated by deformable obstacles, as elastic compliance introduces deformations that are sensitive to the direction of the flow. Third, in the case of deformable obstacles, the effective minimum opening under load is the key control parameter, reflecting the nonlinear balance between hydrodynamic forcing and elastic resistance. Finally, the basic 2D mechanisms for hydraulic asymmetries persist in 3D, although we were unable to reproduce in shark-intestine-inspired geometries the dissipative interactions between primary and secondary flows that are present in Tesla valves and contribute to their hydraulic asymmetry.

Previous quantitative studies of hydraulic asymmetry have largely focused on specific device classes, such as engineered fluidic diodes or individual biological systems. While these approaches have provided valuable insights, their device-specific modeling choices and performance metrics hinder direct comparisons of the roles played by geometry, deformability, and three-dimensionality across systems. Overall, we show that hydraulic asymmetry is primarily controlled by geometry and effective aperture, with material properties influencing the response through the geometries they select under load.

## 2. Materials and Methods

The central quantity used to characterize flow asymmetry is the diodicity *D*, defined as the ratio between the average pressure drops required to generate the same mean flow rate $\bar{Q}$ in the forward ($\Delta P^{+}$) and reverse ($\Delta P^{-}$) directions:

(1) $$D\equiv \frac{\Delta P^{+}}{\Delta P^{-}}\;\mathrm{for}\;a\;\mathrm{prescribed}\;\mathrm{flow}\;\mathrm{rate}\;Q=\bar{Q}.$$

This definition is appropriate when the channel never occludes, as in shark intestines, where a steady flow can be established in both directions. However, biological valves may operate in a regime of complete blockage, where the requirement of sustaining a prescribed flow rate cannot always be satisfied. In such cases, the flow rate in one direction may approach zero and the definition in (1) becomes impractical. It is then more informative to consider the flow rate generated in response to a prescribed pressure drop:

(2) $$Q^{\pm }=Q\left(\Delta P^{\pm }\right),$$

This describes how much flux in the forward $\left(Q^{+}\right)$ and backward $\left(Q^{-}\right)$ direction the channel allows. Full blockage occurs when Q=0 in one direction.

### 2.1. Model Overview

The analysis relies on three numerical models. The baseline configuration is a two-dimensional channel with a single obstacle placed at mid-length, analyzed first as rigid and then as free to deform under hydraulic load. A second two-dimensional model, represents a valve-like configuration in which complete blockage may occur and the alternative description (2) becomes necessary. Both models share the same 2D channel geometry, with length L=80mm and width W=15mm. Finally, three-dimensional geometries are introduced to verify that the mechanisms underlying diodicity persist in higher dimensions and to explore additional effects specific to 3D flows. In this case, the channel is a tubular conduit with diameter D=20mm and length ranging from L=35 to 80mm.

### 2.2. Governing Equations

All simulations were performed in the COMSOL Multiphysics (v6.3; COMSOL AB, Stockholm, Sweden) environment [24]. For the rigid configurations, the geometry is fixed and the flow is governed by the incompressible Navier–Stokes equations:

$$\rho \left(\frac{\partial v}{\partial t}+v\cdot \nabla v\right)=-\nabla p+\mu \nabla^{2}v,\;\nabla \cdot v=0,$$

where v is the velocity field and *p* is the pressure. Water was used as the working fluid, with density $\rho =10^{3}\;\mathrm{kg}/m^{3}$ and dynamic viscosity $\mu =10^{-3}\;\mathrm{Pa}\cdot s$.

When the obstacle is free to deform, the problem becomes fully coupled with a solid domain governed by momentum balance:

$$\nabla \cdot \sigma +b=\rho_{s}\ddot{u},$$

where u is the displacement field, σ is the Cauchy stress tensor, derived from a hyperelastic Neo-Hookean constitutive law, and $\rho_{s}$ is the density of the solid domain. Fluid and solid domains share a common interface where continuity is imposed:

$$v=\dot{u},\;\sigma_{f}n=\sigma_{s}n.$$

with $\sigma_{f}$ and $\sigma_{s}$ representing the fluid and solid stresses, and n the interface normal.

To facilitate comparison across geometries and flow conditions, and with the existing literature, we characterize the flow regime using the Reynolds number,

(3) Re=ρULcμ,

where *U* is the imposed inlet velocity and $L_{c}$ is a characteristic length scale of the channel. In the 2D configurations, $L_{c}$ is taken as the channel width, while in 3D geometries, it is defined as the hydraulic diameter. The ranges of Reynolds numbers explored in each configuration are reported together with the corresponding results.

### 2.3. Study Setup

The boundary conditions follow standard configurations for fluid dynamics problems: a prescribed inlet condition, specifying either a constant uniform velocity $U_{in}$ or a non-zero relative pressure P>0, zero relative pressure at the outlet, and no-slip on all rigid walls. In the deformable cases, the obstacle base has zero prescribed displacement, while the remaining solid boundaries are free to deform under the coupled fluid–structure interaction.

The mesh consists of triangular elements in 2D and of tetrahedral elements in 3D, with rectangular or prismatic boundary-layer elements near the walls, generated by COMSOL Multiphysics (v6.3), using the “physics controlled” meshing option. Mesh motion followed the structural deformation through an ALE formulation, ensuring stable grid quality and solver convergence. Mesh-convergence tests were performed to ensure that the results are independent of the element size. Representative 2D results for a specific test case are reported in Table 1. There, for meshes with number of elements ≥$1.2\times 10^{4}$, the relative deviation on the pressure drop ΔP with respect to the finest mesh remains below 1%. Unless otherwise stated, all 2D simulations reported in this work were performed with ∼$1.2\times 10^{4}$ elements.

The equations were solved with a fully coupled time-dependent solver. Each simulation was run until a quasi-steady regime was reached: either a steady state with negligible temporal variations in pressure drop and flow rate, or a periodic limit cycle. In the latter case, pressure drop and flow rate were post-processed as time-averaged quantities over several oscillation periods.

## 3. Results

**Figure 4:**
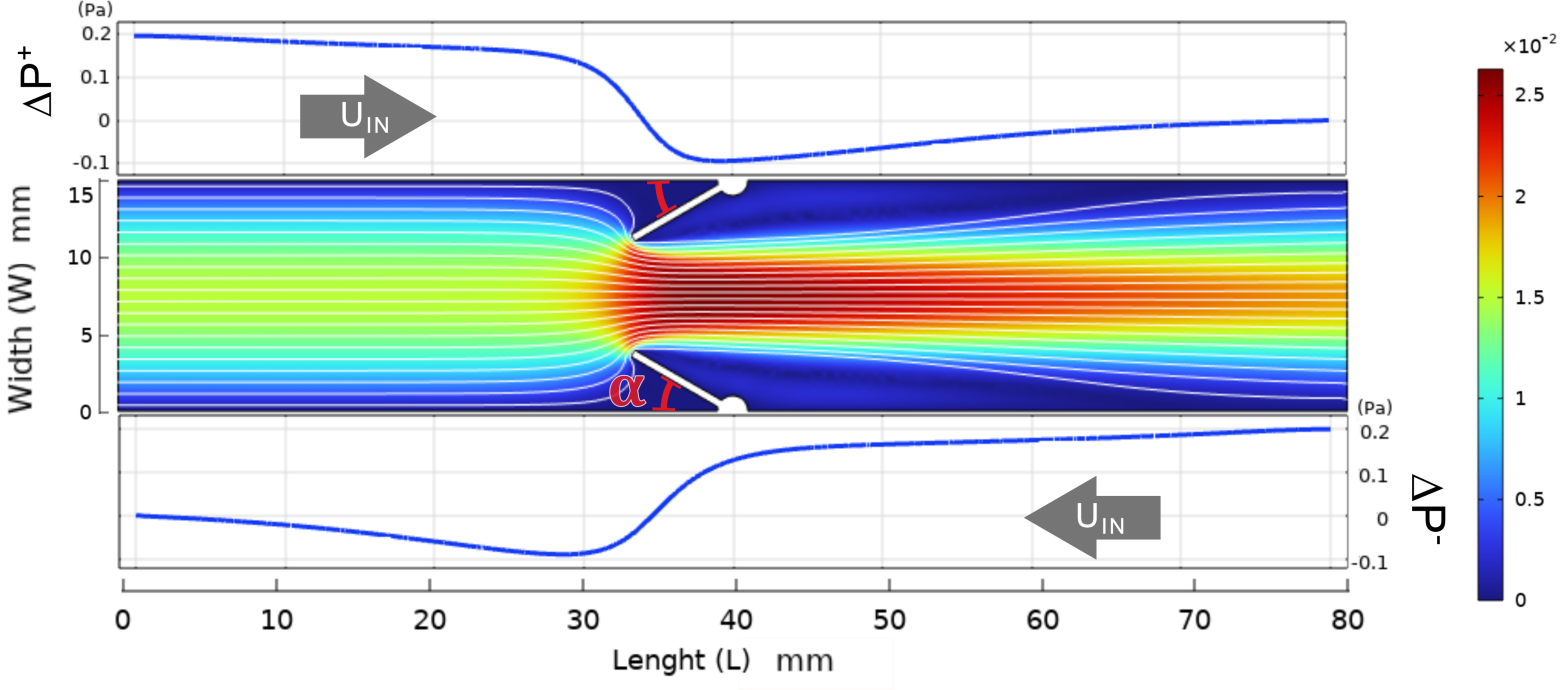
Reference 2D channel used for both rigid and deformable simulations. The obstacle is placed at mid-length and rotated by an angle α. The plots show the pressure along the channel centerline for forward ($\Delta P^{+}$, **top**) and reverse ($\Delta P^{-}$, **bottom**) flow, illustrating the asymmetric pressure distribution and the partial pressure recovery downstream of the obstacle. Color legend indicates velocity magnitude.

We first analyze a simple 2D configuration, used as a baseline to define the forward and backward pressure drops, $\Delta P^{+}$ and $\Delta P^{-}$. Figure 4 shows the reference geometry and the corresponding pressure distributions in the two flow directions.

### 3.1. Two-Dimensional Rigid Channel

**Figure 5:**
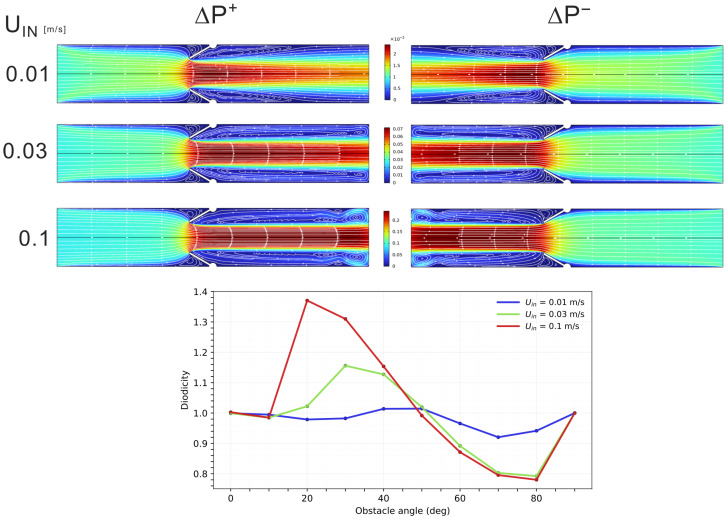
(**Top**) Velocity fields for the 2D rigid channel with α=30° in the forward ($\Delta P^{+}$) and backward ($\Delta P^{-}$) configurations at different inlet velocities. The color legend is shared across rows and indicates the velocity magnitude. (**Bottom**) Diodicity curves D(α) for increasing inlet velocities $U_{in}$, showing higher peak values at larger $U_{in}$.

We vary the obstacle angle α∈[0°,90°] and the inlet velocity $U_{in}\in \left[0.01,0.1\right]\;m/s$. These ranges were selected based on prior parametric tests to cover the regime where diodicity becomes appreciable. Figure 5 shows representative flow fields for α=30° and the plot of diodicity as a function of α.

The diodicity trends can be interpreted by inspecting the recirculation zones in the two directions. Stronger recirculation implies larger viscous and inertial losses: when recirculation is stronger in the backward case, $\Delta P^{-}>\Delta P^{+}$ and D<1. The opposite configuration yields $\Delta P^{+}>\Delta P^{-}$ and D>1. Thus, even a single rigid obstacle is sufficient to produce purely geometric, direction-dependent hydraulic resistance.

### 3.2. Two-Dimensional Deformable Channel

**Figure 6:**
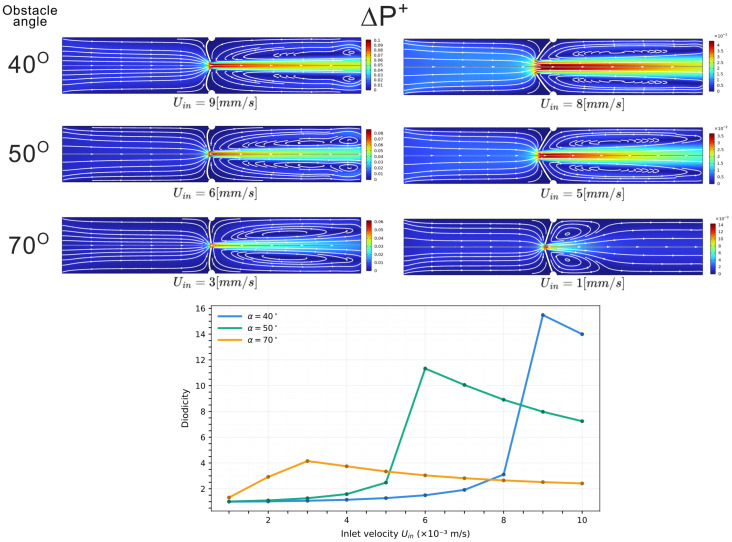
(**Top**) Two-dimensional deformable case, velocity field, and streamlines for forward flow ($\Delta P^{+}$) at fixed elastic modulus $E=10^{4}\;\mathrm{Pa}$. Each row corresponds to a different obstacle angle α, and each column to a different inlet velocity $U_{in}$, leading to different deformation levels and effective apertures. Color bars indicates the velocity magnitude. (**Bottom**) Plot of diodicity as a function of $U_{in}$ and at fixed α, highlighting a peak value at a specific combination of *E*, $U_{in}$, and α where the channel is most obstructed.

The same reference geometry is then considered with the obstacle allowed to deform. We vary the obstacle orientation α∈[0°,90°] and the inlet velocity $U_{in}\in \left[10^{-3},10^{-2}\right]\;m/s$, while keeping the elastic modulus fixed at $E=10^{4}\;\mathrm{Pa}$. These values and ranges were selected based on prior parametric tests to cover the regime where diodicity becomes appreciable. Representative deformed configurations and the corresponding curves showing diodicity as a function of $U_{in}$ and at fixed α are shown in Figure 6.

The diodicity response reflects how the obstacle deforms. In the forward configuration, the obstacle bends into the channel and sharply reduces the cross-section; in the backward case, it bends away and opens the passage. Consequently, a larger pressure drop is required in the forward direction to sustain the same flow rate. A “sweet spot” appears when the deformation produces near-maximal occlusion, causing a sharp peak in diodicity. Small changes in inlet velocity can therefore trigger large reconfigurations of the obstacle and, as a result, strong variations in diodicity.

### 3.3. Annuli Model

**Figure 7:**
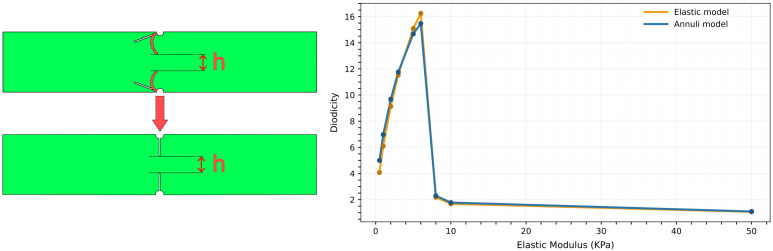
Equivalent annuli model. (**Left**) The original deformed configuration is replaced by a rigid barrier with the same minimum opening *h*. (**Right**) Comparison between diodicity values in the deformable and equivalent rigid cases, showing minor differences and indicating that geometry is the main contributor to the asymmetric effect.

To separate the roles of elasticity and geometry, we introduce a simplified model that mimics the effect of deformation with a rigid obstacle whose opening matches the minimum opening *h* of the deformed geometry between obstacles tips (Figure 7).

We simulate the 2D deformable case at fixed inlet velocity $U_{in}=0.01\;m/s$ and obstacle angle α=30°, varying the elastic modulus in the range E∈[0.5,50]kPa to generate different deformed shapes. This range was selected based on prior parametric tests to span the regime where the deformation-induced minimum aperture (and hence diodicity) becomes appreciable. These shapes are then used to define the opening of the rigid barrier of the equivalent model.

The diodicity values in the deformable and equivalent rigid cases almost overlap, indicating that the dominant contribution does not arise from elasticity itself, but from the geometric reconfiguration generated by deformation.

### 3.4. Complete Blockage Regime

**Figure 8:**
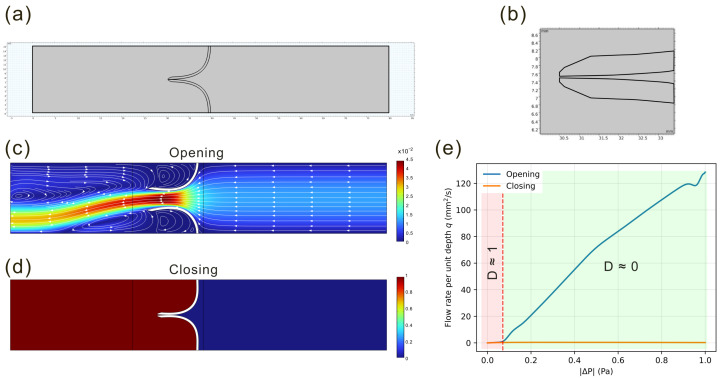
(**a**–**d**) Geometry and elastic response of the leaflet valve. (**a**) Reference configuration with two opposing flexible leaflets; (**b**) zoomed-in view of the valve region showing the small initial gap between the leaflets. (**c**) Velocity magnitude and streamlines in the opening case: the pressure drop separates the leaflets, and a jet crosses the gap and relaxes downstream towards a uniform profile; its slight lateral bending is a symmetry-breaking instability that does not affect the integrated flux. (**d**) Pressure field in the closing case: the reversed pressure pushes the leaflets into contact, forming a barrier and suppressing transport. (**e**) Quasi-steady flow–pressure relation $q(\Delta P^{\pm })$ in both directions. Beyond a small occlusion threshold (red dashed line), the closing branch ($\Delta P^{+}$) remains negligible, while the opening branch ($\Delta P^{-}$) grows almost linearly with $\Delta P^{\pm }$. The shaded bands mark regimes of diodicity *D*: a nearly symmetric response (D≈1) at low load and a complete-blockage regime (D≈0) where the diodic behavior saturates and the valve acts as an almost one-way conduit.

We now consider a bioinspired configuration, taken from the lymphatic system and shown in Figure 8, in which full blockage may occur and the diodicity definition (1) becomes impractical. The model consists of two deformable, leaflet-like obstacles forming a nearly closed channel, with the same wall geometry as in the previous 2D configuration. The elastic modulus is fixed at $E=10^{4}\;\mathrm{Pa}$, chosen to be consistent with pressure levels comparable with those observed in murine lymphatic vessels [1]. When a pressure difference is applied in the forward direction ($\Delta P^{+}$), the two leaflets are pushed against each other, closing the channel. In the reverse direction ($\Delta P^{-}$), the flow separates the leaflets and opens the passage.

As noted in Section 2, we characterize this regime by imposing a pressure difference ΔP∈[0,1]Pa and measuring the resulting flow rates $Q(\Delta P^{\pm })$ in both directions. This range is consistent with values used in modeling physiological lymphatic valves (see [1]), where applied pressure differences are in the order of a few dyne/cm^2^ (e.g., ΔP≈6dyne≈0.6Pa). Since the simulations are performed in 2D, the volumetric flow rate *Q* is obtained by assuming, for simplicity, a unit out-of-plane thickness of 1mm:

(4) Q=q·1mm,

where *q* is the computed planar flux.

In this framework, a symmetric channel would yield comparable flow rates in the two directions for the same pressure drop, $Q\left(\Delta P^{+}\right)\approx Q\left(\Delta P^{-}\right)$. In contrast, a valve-like response is characterized by $Q(\Delta P^{+})\approx 0$ and $Q(\Delta P^{-})>0$ for the same ΔP. Our simulations fall in this latter regime, indicating nearly one-way transport.

### 3.5. Three-Dimensional Channel Results

In this section, we investigate the flow behavior in three-dimensional channels containing rigid and deformable obstacles.

#### 3.5.1. Rigid Cone-Shaped Obstacle

**Figure 9:**
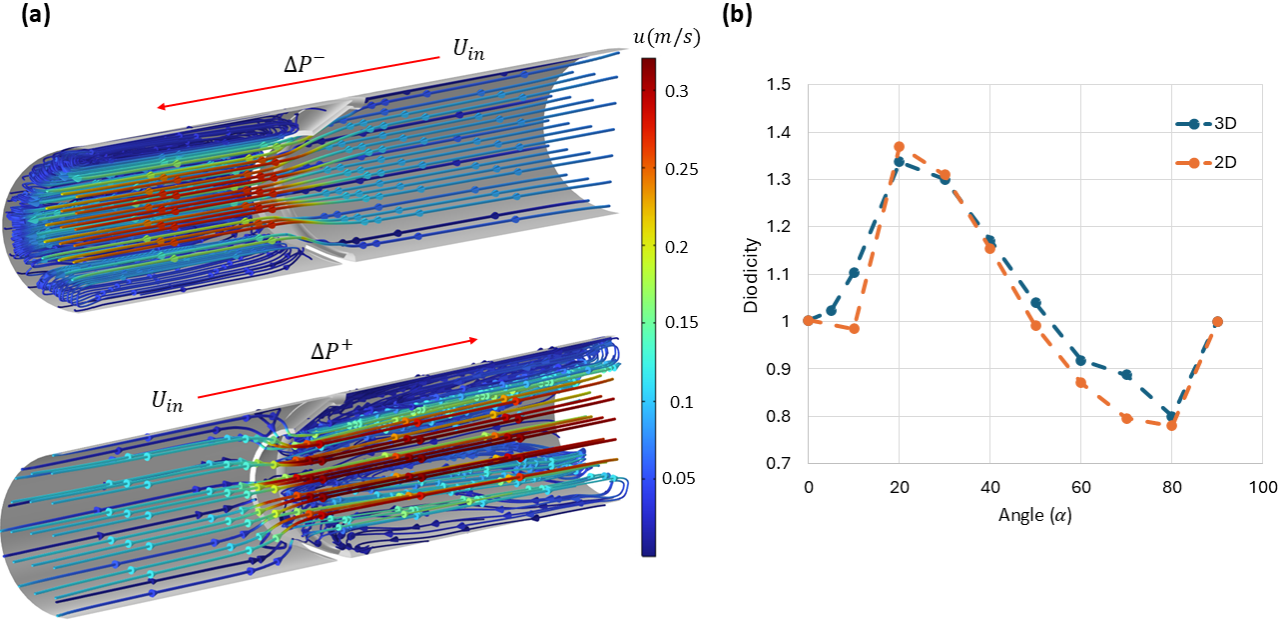
Fluid flow through a 3D channel with a rigid cone-shaped obstruction at a fixed inlet velocity of 0.1 m/s. (**a**) Streamlines for flow in opposite directions; the color bar indicates the magnitude of the velocity field. For clarity, only half of the channel is shown. (**b**) Diodicity values calculated for various obstacle angles, compared with the 2D simulations in Figure 5.

We first analyzed how the angle of the obstacle influences the diodicity of a channel equipped with a rigid cone-shaped obstacle. This 3D geometry was constructed by revolving the profile of the corresponding 2D channel about its axis. In all simulations, a constant inlet velocity of 0.1m/s was imposed to match the 2D boundary conditions and enable direct comparisons across models. Figure 9a presents the flow streamlines in both directions for the 3D channel with a cone angle of α=20°. Figure 9b shows the diodicity as a function of obstacle angle for both the 3D and 2D simulations. Although there are discrepancies at some angles between the two sets of results, the overall trend from the 2D simulations aligns well with the 3D data.

#### 3.5.2. Rigid and Deformable Incomplete Cone-Shaped Obstacles

**Figure 10:**
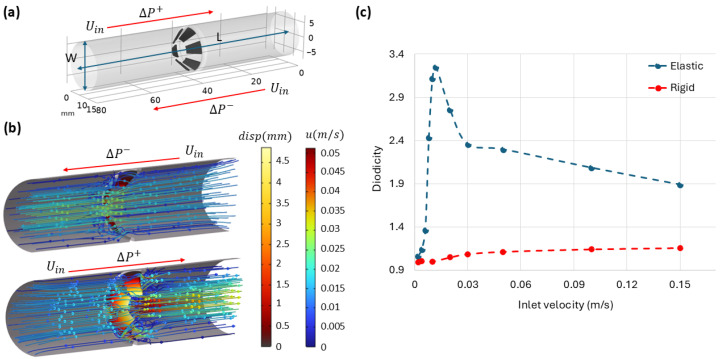
Fluid flow through 3D channels with rigid and deformable incomplete cone-shaped obstruction for a fixed angle and various inlet velocities. (**a**) Geometry of the 3D channel with an incomplete cone-shaped obstacle composed of six leaflets. The pressure drops for forward and backward flow are denoted by $\Delta P^{+}$ and $\Delta P^{-}$, respectively. (**b**) Streamlines for flow in both directions through the channel with a deformable obstacle, along with the corresponding deformation under fluid force. The color bars indicates the displacement magnitude (disp) of the obstacle and the magnitude of the fluid velocity (*u*). For clarity, only half of the channel is shown. (**c**) Computed diodicity for channels with both elastic (*E* = 1 kPa) and rigid obstacles over different inlet velocities.

To explore the mechanism responsible for the strong diodicity observed in channels with 2D deformable obstacles, we developed a 3D model consisting of an incomplete cone made of six leaflets (Figure 10a). The geometry was optimized to avoid buckling and contact between the leaflets during deformation. The obstacle’s initial opening angle was set to α=40°, and its elastic modulus was set to *E* = 1 kPa. As shown in Figure 10c, the channels with these incomplete cone obstacles exhibit a significant increase in diodicity in the deformable case compared with the rigid one, particularly when the opening area decreases markedly for forward ($\Delta P^{+}$) flow (Figure 10b) under an inlet velocity of 0.012 m/s.

It is worth noting that the maximum diodicity obtained in this 3D configuration is around 3.3, which is notably smaller than the maximum value observed in 2D simulations. This difference arises because, in the 3D simulations, finite gaps always remain between the leaflets, allowing some fluid to pass through even when the obstacle deforms. Nevertheless, these results confirm that obstacle elasticity and the reduction in the effective opening area are key factors in enhancing diodicity. These observations are fully consistent with the mechanisms identified in the 2D simulations.

#### 3.5.3. Shark-Intestine-Inspired Obstacles

**Figure 11:**
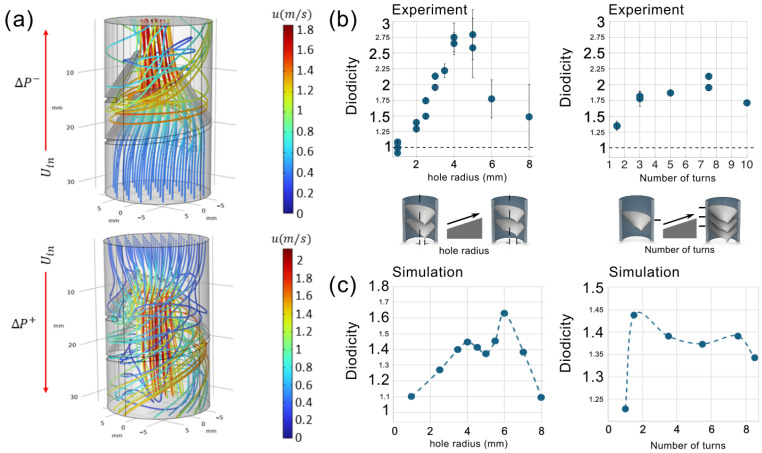
Fluid flow through 3D channels with a rigid spiral obstacle inspired by the shark intestine. (**a**) Streamline patterns for flow in both directions. Numerical and experimental [2] diodicity values for spiral obstacles with (**b**) various inner hole radii and (**c**) different numbers of turns.

Lastly, we examined flow asymmetry in channels with rigid spiral obstacles inspired by the structure of the shark intestine. The geometries were constructed based on the experimental study in [2]. The inlet velocity was kept constant at 0.38 m/s. Figure 11a shows the flow streamlines in both directions, highlighting the inherent asymmetry in the fluid motion. We then performed a parametric analysis of the flow in both the top-to-bottom and bottom-to-top directions, varying the inner radius of the spiral obstacle as well as the number of turns. Figure 11b,c, compare the computed numerical diodicities for various inner radii and numbers of turns, with the experimental results reported in [2]. As shown, the numerical simulations confirm the presence of flow asymmetry in channels with rigid spiral obstacles, and the overall trends align reasonably well with the experimental observations.

**Figure 12:**
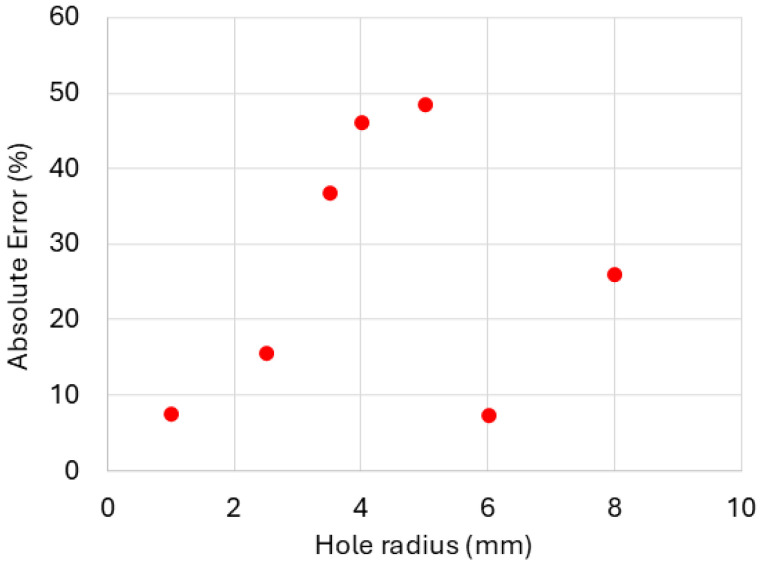
Absolute percentage error in diodicity for 3D channel with spiral obstacle obtained from numerical simulations relative to experimental measurements for different inner hole radii.

**Figure 13:**
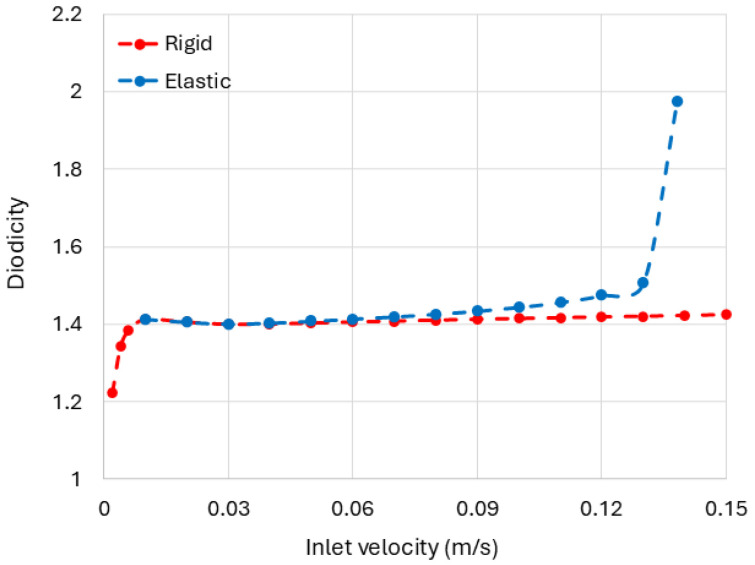
Computed diodicity for channels with both elastic (*E* = 1 MPa) and rigid spiral shark-intestine-inspired obstacles over different inlet velocities. In these simulations, the number of turns is 1.5, the inner radius is 3 mm, and pitch distance is 7.5 mm.

However, some discrepancies in the predicted diodicity values can be observed. Figure 12 shows the absolute percentage error in diodicity for various hole radii for the 3D channel with a spiral obstacle. We attribute the larger error values in the central region of the diagram to mismatches associated with uncertainties in the experimental setup. In fact, we are able to see diodicity values approaching 2 and beyond only by invoking the deformability of the spiral obstacle (see Figure 13), while for the perfectly rigid case, we could not exceed the value of 1.65. The larger errors correspond precisely to the regime in which we underestimate the diodicity with our perfectly rigid obstacle. Even small deformability would shift the predictions to higher values, reducing the error with respect to the experiment.

We also performed a series of simulations to investigate how obstacle deformability affects diodicity in channels with spiral obstructions. In these simulations, the number of turns was fixed at 1.5, the pitch distance at 7.5 mm, and the inner radius at 3 mm, while the inlet velocity was varied. The elastic modulus of the obstacle was set to *E* = 1 MPa. Figure 13 shows that the elastic behavior of the obstacle increases the diodicity compared with the rigid case as the inlet velocity is increased. At higher inlet velocities, however, the simulations experienced convergence challenges due to contact interactions and structural instabilities within the deformable spiral. As a result, diodicity values could not be obtained in this regime using the current modeling framework. Addressing these limitations will require more advanced numerical techniques and is left for future work.

## 4. Discussion

The configurations analyzed in this work show that hydraulic asymmetry in tubular channels can be interpreted within a single geometric framework. Hydraulic asymmetry emerges when reversing the flow direction reorganizes the topology of streamlines. This cannot occur in the case of flows in undeformable domains with fore-aft symmetry, or in the limit of vanishing Reynolds numbers, because of symmetries in the governing equations. In the presence of elastic obstacles, the flow reshapes the channel’s effective aperture in a direction-dependent manner. The examples discussed in the Introduction can thus be revisited under this unified lens.

In the 2D rigid case, asymmetry arises from geometric steering alone. Reversing the flow direction reorganizes streamline topology, as well as inertial and viscous losses. Then different pressure drops, $\Delta P^{+}$ and $\Delta P^{-}$, are required to maintain the same mean flow rate. This mechanism is well documented in the literature on Tesla-type valves through detailed numerical and experimental investigation. We use these results as a quantitative benchmark for passive fluidic diodes. Here, however, we consider a markedly different setting: a single-stage channel with an obstacle inspired by biological architectures. Despite the absence of design optimization, our results show that such geometric bias already produces a measurable diodicity, without moving parts or material nonlinearity. In our simulations, inlet velocities of $U_{\mathrm{in}}$ = 0.01–0.1 m/s yield peak diodicity values in the range D≃ 1.01–1.37, corresponding to Reynolds numbers of Re≃ 150–1500. Although smaller than those reported for optimized multistage Tesla valves (D∼1.8–2.3 at comparable Reynolds numbers [15]), these values demonstrate that geometric steering alone can already produce a significant directional bias, in geometries much simpler than Tesla’s.

In the 2D deformable channel, forward and backward flow bend the obstacle toward or away from the main stream path, narrowing or reopening the passage. Replacing the deformed obstacle with an equivalent rigid barrier with the same minimum opening *h* leaves the diodicity almost unchanged. This shows that the key quantity is the aperture selected under load. This single parameter captures the competition between hydrodynamic forces and elastic resistance, which is strongly nonlinear. As a consequence, small variations in inlet velocity can trigger abrupt changes in the selected aperture, producing sharp peaks in the diodicity curves. In our simulations, the peak diodicity value reaches D≃15.5 at a Reynolds number of Re≃135. By comparison, numerical studies on Tesla-type valves report peak values around D≃4.4 at Re∼300, and lower diodicities at Reynolds numbers comparable to those explored here [15].

The bioinspired valve configuration extends this mechanism to a regime in which the passage is almost completely closed in one direction while remaining open in the other. The same principle underlies physiological valves in the heart and lymphatic system, where backflow is suppressed in an analogous manner. Our valve-like simulations reproduce this behavior.

**Figure 14:**
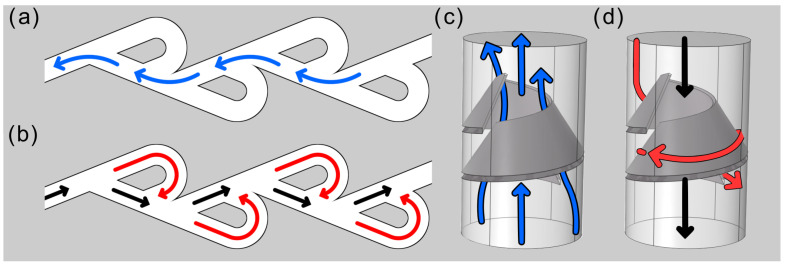
Conceptual comparison between the Tesla-valve principle and the shark-intestine-inspired geometry. Schematic flow pathways highlight how geometric features generate secondary streams (red) that interact with the primary through-flow (black). Flow in the opposite direction (blue) is mainly undisturbed by secondary flows. Tesla-valve configuration (**a**,**b**) promotes pronounced secondary recirculation. Shark-intestine-inspired geometry (**c**,**d**) yields weaker disturbance of primary flow by secondary streams.

Three-dimensional simulations confirm that hydraulic asymmetry persists in fully three-dimensional geometries. For incomplete conical obstacles, the diodicity values attainable in 3D are lower than their 2D counterparts, as the maximum achievable asymmetry is limited by finite gaps. In shark-intestine-inspired obstacles, our 3D rigid simulations capture the experimentally observed trends as the inner radius and the number of turns vary. Introducing deformability further enhances diodicity, although numerical issues associated with contact restrict the range of velocities that we could explore. Previous numerical studies on Tesla valves have shown that turbulence models can improve quantitative agreement with experiments at moderate Reynolds numbers (Re∼500–2000) [15,25]. At higher Reynolds numbers (Re∼7600), our preliminary tests using a RANS *k*–ε model in our more complex spiral geometry do not lead to significant changes in the computed values of diodicity. A detailed turbulence-model sensitivity analysis is left for future work. Our analysis suggests that the 3D design based on an internal helical obstacle, loosely inspired by the anatomy of the shark intestine, does not reproduce one of the key mechanisms that generate hydraulic asymmetry in classical 2D Tesla valves. This is illustrated in Figure 14.

Indeed, in the Tesla valve case, the conflict between primary and secondary streams arising in the backward flow direction (black and red streamlines, respectively) leads to larger hydrodynamic losses than in the forward flow direction (blue streamlines) and, hence, to potentially large values of the diodicity. By contrast, in the case of the 3D shark-intestine-inspired helical structure, the primary and secondary streams in the backward direction have components along the tube axis with the same sign. Thus, the secondary stream induces smaller hydrodynamic disturbance of the primary stream, and lower hydrodynamic losses with respect to the 2D Tesla scenario. It is left to future investigations to explore whether by fine tuning the 3D obstacle geometry (e.g., by considering different ratios of pitch, external radius, or internal hole radius, or by reproducing the shark anatomy more faithfully) or, alternatively, by exploring 3D geometries departing more drastically from the shapes bio-inspired by the shark intestine anatomy, one may arrive at diodicity values larger than the ones we have found in our study.

From a design viewpoint, the primary control parameter is the effective minimum opening selected under load. For a given operating range of flow speed *U* and obstacle angle α, our models (Section 3.3) identify stiffness values *E* that yield the smallest selected aperture and therefore maximize hydraulic asymmetry. Conversely (Section 3.2), in deformable-obstacle simulations at fixed *E*, varying *U* and α controls the selected aperture and determines whether the response remains weak or becomes strongly asymmetric. The triplet (U,α,E) therefore defines a compact design space that links driving, geometry, and stiffness to the selected minimum opening. The same parameter space applies to valve-like operation, where performance is expressed in terms of flow rate rather than hydraulic asymmetry.

This framework can be used as a practical design map. In soft-valve concepts, we can tune *E* so that physiological pressure loads induce markedly different deformations (and thus different minimum openings) in forward versus reverse flow. In rigid devices, we can select (U,α) in the regimes where directional recirculation is most pronounced to obtain robust baseline asymmetry. Overall, this approach puts non-occluding and valve-like architectures on the same footing and provides practical guidelines to design passive soft devices for directional flow control.

## Figures and Tables

**Table 1:** Representative 2D mesh-convergence study for the rigid obstacle at α=50°, monitoring the pressure drop ΔP. The finest mesh (number of elements = 617,898) is used as a reference to compute the relative error.

#	Elements	Δ*P*	Rel. Err.
1	2506	0.91976	3.17%
2	4734	0.92852	2.25%
3	7006	0.93617	1.44%
4	12,	0.94484	0.53%
5	28,130	0.94602	0.40%
6	66,728	0.94830	0.16%
7	118,952	0.94885	0.11%
8	617,898	0.94986	0.00%

## Data Availability

The data that support the findings of this study are available from the corresponding author upon reasonable request.
